# A Neuropsychiatric Assessment of Children with Previous SARS-CoV-2 Infection

**DOI:** 10.3390/jcm12123917

**Published:** 2023-06-08

**Authors:** Veronica Scarselli, Dario Calderoni, Arianna Terrinoni, Chiara Davico, Giulia Pruccoli, Marco Denina, Chiara Carducci, Andrea Smarrazzo, Melania Martucci, Mariaelena Presicce, Daniele Marcotulli, Luca Arletti, Mauro Ferrara, Silvia Garazzino, Rosanna Mariani, Andrea Campana, Benedetto Vitiello

**Affiliations:** 1 Department of Human Neuroscience, Sapienza University of Rome, 00185 Rome, Italy; 2 Department of Public Health and Pediatric Sciences, University of Turin, 10126 Turin, Italy; 3 Emergency, Acceptance and General Pediatrics, Bambino Gesù Children’s Hospital, 00165 Rome, Italy

**Keywords:** COVID-19, SARS-CoV-2, long-COVID, children, adolescents

## Abstract

Aim: Concerns have been raised about possible neuropsychiatric sequelae of COVID-19. The objective of this study was to examine the plausibility of long-term mental health consequences of COVID-19 by assessing a sample of children after the resolution of the acute SARS-CoV-2 infection. Method: As part of a systematic follow-up assessment of pediatric patients with COVID-19 conducted at two university children’s hospitals, 50 children (56% males) aged 8 to 17 years (median 11.5), 26% with previous multisystem inflammatory syndrome in children (MIS-C), without a prior history of neuropsychiatric disorders, received a battery of clinical neuropsychiatric and neuropsychological rating scales that included the Pediatric Migraine Disability Assessment (PedMIDAS), Sleep Disturbance Scale for Children (SDSC), Multidimensional Anxiety Scale for Children (MASC-2), Child Depression Inventory (CDI-2), Child Behavior Checklist (CBCL), and the NEPSY II (Neuropsychological Assessment, Second Edition). The assessments were conducted between 1 and 18 months (median 8 months) after the acute infection. Results: The CBCL internalizing symptoms score was in the clinical range for 40% of the participants (vs. a population expected rate of about 10%, *p* < 0.001). A sleep disturbance was detected in 28%, clinically significant anxiety in 48%, and depressive symptoms in 16%. The NEPSY II scores showed impairment in attention and other executive functions in 52%, and memory deficits in 40% of the children. Conclusions: These data from direct assessment of a sample of children who had SARS-CoV-2 infection show higher than expected rates of neuropsychiatric symptoms, thus supporting the possibility that COVID-19 may have mental health sequelae long after the resolution of the acute infection.

## 1. Introduction

Since the COVID-19 pandemic began, reports of a possible involvement of the nervous system have increasingly emerged [1]. A retrospective study of a clinical sample of infected patients in Wuhan reported a 36% prevalence of neurological symptoms [2]. A number of psychiatric manifestations, including anxiety, mood disorders, and suicidal ideation were found in a large sample of adults with COVID-19 [3]. In children, SARS-CoV-2 infection usually presents with mild to moderate symptoms, most commonly fatigue, headache, and anosmia, with a median duration of 6 days [4]. However, a 2% rate of admission to pediatric intensive care unit (ICU) has been reported, with about 1% presenting a post-infectious hyper-inflammatory picture known as multisystem inflammatory syndrome in children (MIS-C) [5]. In pediatric patients hospitalized for COVID-19, 22% were found to have neurologic involvement, including transient symptoms, severe encephalopathy, stroke, central nervous system infection/demyelination, Guillain-Barré syndrome/variants, and acute fulminant cerebral edema [6].

Concerns have been raised that COVID-19 may have long-term persistent symptoms of various nature, a condition being referred to as *long COVID* [7,8]. In a 12-month follow-up study, non-hospitalized children aged 11–17 years, who were either SARS-CoV-2 positive or negative, completed an online health questionnaire. While the symptoms present at the time of the acute COVID-19 declined over time, a few children reported new symptoms, mainly shortness of breath and fatigue, which persisted for several months [9]. Another survey, with an online questionnaire of a large cohort of children aged 0–17 years, found that SARS-CoV-2 positive children reported symptoms, such as fatigue, muscle weakness, and dizziness, more frequently than SARS-CoV-2 negative peers, with symptom resolution in most cases within 1–5 months [10]. A national population online survey study of adolescents in Denmark found that SARS-CoV-2 positive youth were more likely to report at least one persistent symptom (e.g., dizziness, chest pain, breathing difficulties) than uninfected peers, even though the latter reported worse quality of life, possibly due to pre-existing comorbidities [11].

In adults, neuropsychiatric symptoms have been reported after the resolution of the COVID-19 acute phase, including fatigue, anxiety, depression, psychotic symptoms, and deficits of attention and memory, with variable prevalence across studies [12]. In a large study, 33% of the participants were found to have one or more neurological or psychiatric diagnoses in the 6 months following the resolution of acute COVID-19 [13]. A clinical sample of 38 adults hospitalized for complications of SARS-CoV-2 infection underwent neuropsychological testing about 5 months after hospital discharge; the results showed that 42.1% of the sample had processing speed deficits, 26.3% deficits in delayed verbal recall, and 21% deficits in both processing speed and verbal memory [14]. 

Few studies have specifically examined possible neuropsychiatric sequelae of COVID-19 in children [15]. In a large sample of children who were prospectively followed for up to 12 months after the SARS-CoV-2 infection, the most commonly reported symptoms were fatigue (19%), headache (12%), insomnia (7.5%), muscle pain (6.9%), and problems with concentration (6.8%) [16]. In a case-control study of 25 children aged 12 to 17 years, significant impairment was found in memory, attention, visual gnosis, visual-spatial function, kinesthetic and dynamic praxis, and verbal and non-verbal components of thinking both 1–2 weeks after the acute phase and 2–12 months later [17]. A recent narrative review has reported that anxiety, sleep problems, and deficits in memory and concentration are the main long-term complaints in children after COVID-19 [18].

At this time, while progress has been made in the management of the acute phase of the SARS-CoV-2 infection, the possible neuropsychiatric manifestations of long COVID in children remain poorly understood and understudied, with current data being mainly impressionistic or based on online surveys or parental report rather than on actual examination of patients.

The objective of this study was to examine the plausibility of neuropsychiatric sequelae of COVID-19 in children (here defined as subjects under 18 years of age) by systematically assessing the prevalence of neurological symptoms (headache, sleep disturbances), neuropsychological impairment (attention, memory deficits), and specific psychiatric symptoms (mood problems, anxiety) in a sample of pediatric patients, who had no evidence of previous neuropsychiatric disturbances, months after the resolution of the acute SARS-CoV-2 infection. While the absence of pre-COVID-19 assessments in the children participating in this study prevents drawing causal inferences, the rate of neuropsychiatric symptoms can provide support to the concept of a neuropsychiatric long COVID in children, when compared with the expected population rates that had been observed prior to the COVID-19 pandemic. We used validated instruments to assess psychopathology, with expected rates of clinically significant symptoms below 10% in the child population [19,20,21]. Thus, a previous epidemiological study in the Italian population had reported a prevalence of clinically significant scores on the Child Behavior Checklist (CBCL) of 9.8% (95% C.I. 8.8–10.8) [19]. As a reference, the worldwide prevalence of mental disturbances in children and adolescents had been estimated at 13.4% (95% C.I. 13.3–15.9) for any disorder, 6.5% (4.7–9.1) for anxiety, and 2.6% (1.7–3.9) for depressive disorders [22]. A secondary aim was to explore possible demographic and clinical predictors of neuropsychiatric symptoms.

## 2. Materials and Methods

### 2.1. Design

This was a prospective evaluation of children with a previous SARS-CoV-2 infection, who were assessed at two university pediatric hospitals in Italy (Bambino Gesù Hospital in Rome and Regina Margherita Children’s Hospital in Turin) from March 2020 to September 2021. The study specific assessments were collected between July and November 2021. Our clinical sample is composed of the children who have made several day hospital visits after COVID-19 infection to check for any cardiac, general pediatric, and pulmonary sequelae. The neuropsychiatric assessment was part of a general medical follow-up, with pediatric, cardiac, and pulmonary assessments, conducted in a day hospital setting. 

The study was conducted according to the ethical standards and principles regulating clinical investigations and was consistent with the Helsinki Declaration of 1975, as revised in 2008. The study protocol was approved by the institutional ethics committee. 

### 2.2. Participants

Study participants were enrolled based on the following inclusion criteria: (1) age between 8 and 17 years; (2) SARS-CoV-2 infection confirmed by positive real-time reverse-transcriptase polymerase chain reaction (RT-PCR) from a nasopharyngeal and/or throat swab or finding of specific antibodies in case of MIS-C; and (3) parental written informed permission to participate. Children who had received a neuropsychiatric diagnosis prior to the date of the SARS-CoV-2 infection were excluded. 

### 2.3. Assessments

Demographics and clinical data were collected, including age, sex, symptoms during the acute phase of the infection, post-COVID medical sequelae, and, in case of hospitalization for COVID-19, duration of the inpatient stay. For all participants, a careful medical history was obtained to exclude previous neuropsychiatric evaluations and diagnoses of neurologic and psychiatric disorders. 

The neuropsychiatric assessment included:

(1) A standard neurological examination, carried out by medical doctors.

(2) The Pediatric Migraine Disability Assessment (PedMIDAS), Italian standardized version, which is a validated questionnaire completed by the child for the assessment of pediatric headache. The first three questions are about the impact of headache at school: question 1 asks about school day absences; question 2 asks about partial day absences; and question 3 asks about functioning at 50% or less ability in school. The fourth question assesses the impact of headache at home, including inability to perform homework and chores. The final two questions assess disability in social functioning including sports; question 5 asks about complete absence from activities, while question 6 asks about functioning at 50% or less of their ability. A raw score is obtained by adding the six individual questions. A score between 0 and 10 indicates grade 1 (disability none/mild), a score between 11 and 30 (grade 2/medium disability), a score between 31–50 (grade 3/moderate disability), a score >50 (grade 4/severe disability) [23]. 

(3) The Sleep Disturbance Scale for Children (SDSC), Italian standardized version, which is a questionnaire completed by parents. It examines possible disturbances in sleep initiation and maintenance, sleep breathing, arousal, sleep-wake transition, excessive sleepiness, and hyperhidrosis. [24]. The SDSC uses a normative sample to produce standard T scores in order to compare the raw scores to the average population scores. T score average is 50 with a standard deviation of 10. Cut off for clinical significance was set at >70 corresponding to >95th percentile. 

(4) The Multidimensional Anxiety Scale for Children–2nd edition (MASC-2), Italian standardized version, a self-rated questionnaire completed by the child to assess anxiety symptoms [25]. The MASC-2 uses a normative sample to produce standard T scores in order to compare the raw scores to the average population scores (matched to age and sex). Cut off for clinical significance was set at >65 (corresponding to >92nd percentile of the population).

(5) The Child Depression Inventory, 2nd edition (CDI-2), Italian standardized version, which is completed by the child and assesses age-specific manifestations of depressive symptoms [26]. The CDI-2 uses a normative sample to produce standard T scores in order to compare the raw scores to the average population scores (matched to age and sex). Cut off for clinical significance was set at >65 (corresponding to >92nd percentile of the population).

(6) The Child Behavior Checklist (CBCL), Italian standardized version, which is completed by the parent and assesses emotional and behavioral problems [27]. The CBCL uses a normative sample to produce standard T scores from comparing the raw scores to the average population scores (matched to age and sex). T score average is 50 with a standard deviation of 10. Cut off for clinical significance was set at >70, corresponding to scores above the 92nd percentile.

(7) The developmental NEuroPSYchological Assessment (NEPSY-II), Italian standardized version, which is a battery of tests administered to the child about attention, executive functions, and immediate memory, assessing the ability to inhibit automatic responses, selective and sustained attention, immediate verbal memory, and visuospatial memory. More specifically, six tests make up the domain of Attention and Executive Functions. The *Visual Attention* test assesses visual scanning and selective visual attention abilities. The *Design Fluency* test assesses behavioral productivity. The participant is asked to generate unique designs by connecting up to five dots presented in a structured or random array. In the *Auditory Attention and Response Set* test, the participant listens to a series of words and points to the appropriate colored circle when they hear a target word. *Auditory Attention* evaluates selective and sustained auditory attention. *Response Set* assesses the ability to shift and maintain a new, complex response set involving the inhibition of the automatic response and alternating between matching or contrasting stimuli. *Inhibition* evaluates the ability to inhibit automatic responses and to shift between congruent or incongruent responses during the naming of visual stimuli. Concerning memory assessment: *Word List Interference* assesses verbal working memory, repetition, and word recall following interference. The *Memory for Designs* test assesses memory for the form and spatial location of novel visual material. *List Memory* assesses long-term verbal memory for lists of words. T score average is 10. Cut off for clinical significance was set at 2 standard deviations from the average score of the population [28]. 

### 2.4. Statistical Analyses

Statistical analyses were performed using the statistical programming language R (version 4.0.5) (47). Descriptive statistics were applied to the sociodemographic and clinical data. Continuous variables were described by means and SD, and categorical data as percentages. The main outcomes variables included presence of anxiety (MASC-2), depressive symptoms (CDI-2), sleep disturbances (SDSC), and the NEPSY-II scores for graphic fluency and visual attention. Separate regression models were used to identify variables associated with each outcome. All regression models included the following regressors as independent variables: sex (as factor), age (years), hospitalization (as factor), a diagnosis of MIS-C (as factor), time (duration of the follow-up, i.e., days from the acute COVID-19 until the assessment time), and the headache-related disability as evaluated by the PedMIDAS score. Since the NEPSY-II subscales can be inter-correlated, a multivariate linear regression was used to determine the association of the considered regressors (i.e., sex, age, hospitalization, MIS-C diagnosis, and PedMIDAS) with executive functions impairment (i.e., the NEPSY-II scores for graphic fluency and visual attention). The R^2^ were computed to examine the validity of the regression models. The models were presented even if the R^2^ were low, to document that the variance in the outcome was not adequately accounted for by the available regressors. Pillai’s trace statistic was used to evaluate significance of the multivariate model, the associated univariate multivariable effects were then analyzed, and *p*-values corrected using the Benjamini–Hochberg procedure. Normality of the residuals was assessed with the Shapiro–Wilk test, which showed violation of normality for the CDI and MASC data. We conducted rankbased estimation of the models with the same regressors and outcomes. In all statistical analyses performed, *p* < 0.05 was considered to be statistically significant.

## 3. Results

### 3.1. Sample Demographics and Clinical Characteristics

Of 64 children who were invited to participate, 52 met the study inclusion criteria and 50 agreed to participate. The study sample consisted of 28 males (56%) and 22 females (44%), aged between 8 and 17.11 years (median 11.5 years) (Table 1). Of them, 35 had been hospitalized (70%) due to severe symptoms, while the remaining 15 patients (30%) had been treated at home. A total of 13 (26%) had been hospitalized because of MIS-C, 13 (26%) had received oxygen therapy, and 5 (10%) had been treated in an Intensive Care Unit (ICU). 

The symptoms most frequently presented during the acute phase were fever (n = 32; 70%), cough (n = 13; 26%), sore throat (n = 13; 26%), abdominal pain (n = 13; 26%), skin rash (n = 12; 24%), conjunctivitis (n = 11; 22%), headache (n = 11; 22%), vomiting (n = 11; 22%), diarrhea (n = 10: 20%), asthenia (n = 10; 20%), and dyspnea (n = 10; 20%) (Table 1). 

### 3.2. Neuropsychiatric Follow-Up Assessments

The neuropsychiatric evaluation was conducted between 1 and 18 months (median 8 months) after the acute infection. Among the clinically reported symptoms present at the time of the assessment, there were: asthenia (n = 10), inattention, concentration, and memory problems especially evident in school (n = 7), headache (n = 8), sleep difficulties (n = 2), and persistent ageusia and anosmia (n = 1). 

On the PedMIDAS, 5 children (10%) had mild functioning impairment associated with headache and one child (2%) complained of moderate disability associated with headache.

#### 3.2.1. Neurological Assessment

Neurological physical examination: 10 patients (20%) showed minimal and non-specific abnormalities at the neurological physical examination, such as tremor or coordination difficulties.

#### 3.2.2. Sleep

On the SDSC, 14 patients (28%) had a total score in the clinical range. The most frequently reported problems were Disorders of Excessive Somnolence (DES) (n = 15; 30%) and Disorders of Initiating and Maintaining Sleep (14) (n = 13; 26%) (Table 2).

#### 3.2.3. Psychiatric Assessment

On CDI-2, 8 children (16%) presented total scores in the borderline or clinical range. The multivariable regression model exploring possible predictors of depression (CDI-2 total score) showed no association of the CDI-2 scores with age, sex, history of hospitalization, headache, migraine, or length of follow-up (Table 3).

On the MASC-2, 24 children (48%) had a total score above the cut-off of 65, indicative of significant anxiety symptoms. The most frequently reported anxiety symptoms were public performance fears (n = 28; 56%), separation anxiety (n = 26; 52%) and harm avoidance (n = 25; 50%). Moreover, there were scores above the normal cut-off for the following subscales: Generalized Anxiety Disorder (n = 23; 46%), Social Anxiety (n = 21; 42%), Panic (n = 21; 42%), and Obsessions and Compulsions (n = 20; 40%). The multivariable linear regression model exploring possible predictors of anxiety (MASC-2 total score) showed no association with age, sex, history of hospitalization, headache, migraine, or length of follow-up. History of MIS-C was associated with lower anxiety levels [estimated mean difference = -9.91 (95% CI: −18.30 to −1.51), Table 3]. 

On the CBCL, 20 children (40%) had abnormal internalizing problems scores (i.e., above the cut-off of 65), more specifically in the dimensions of Anxiety (n = 13; 26%), Somatic Complaints (n = 12; 24%), and Affective Problems (n = 11; 22%). The observed rate (40%) is greater than the expected from population norms (10%, *p* < 0.0001). The multivariable linear regression model did not show any association of the CBCL total internalizing score with sex, age, hospitalization, MIS-C, headache (PedMIDAS score), or length of follow-up (Table 3).

#### 3.2.4. Neurocognitive Assessment

The NEPSY-II showed a neuropsychological performance below that expected for age for visual attention (n = 20; 40%), auditory attention (n = 26; 52%), and response set item (n = 30; 60%). 

In the design fluency item, which specifically evaluates sustained attention and the capacity for cognitive flexibility, planning, and monitoring, half of the children had scores below the norm (n = 25; 50%). Difficulties in the ability to inhibit automatic responses were found in 40% of the children. Scores were in the borderline or clinical range for the drawing memory test (visual-spatial memory) in n = 20 (40%) and for the list memory item (verbal memory) in n = 25 (50%).

In multivariable regression analyses, no association was found between sex, age, hospitalization, MIS-C, migraine, or length of follow-up, and the NEPSY scores for attention or graphic fluency (Table 3).

## 4. Discussion

This prospective follow-up of children after acute COVID-19 found rates of depressive and anxiety symptoms and of executive functions deficits that were higher than expected, according to population norms. These children did not have a pre-COVID-19 history of neuropsychiatric disorders nor were they known for having cognitive or behavioral problems. The lack of pre-COVID assessments prevents making within subject pre-post comparisons, and the lack of a matched control without COVID-19 does not allow between group comparisons to be assessed. For these reasons, no causal inferences can be drawn from the data. The results, however, are compatible with the presence of neuropsychiatric long-term sequelae of COVID-19 with mood and anxiety symptoms, and deficits in attention, executive functions, and memory. 

The neurological examination did not reveal any clinically significant signs. Headache was reported by about one out of the six children, with mild to moderate impact on daily life. Sleep disturbance, most commonly excessive sleepiness, was reported for about one fourth of the children. These findings are consistent with previous reports in both adults and children after acute COVID-19 [13,17]. In adults, cognitive impairment was recorded lasting more than one year after the acute infection [29]. 

The psychiatric assessments evidenced predominantly anxiety symptoms, affecting about half of the sample, while depressive symptoms were less common, being detected in about a sixth of the sample. It should be noted that these data referred to a period characterized by considerable uncertainty, which can explain an immediate experience of anxiety reactive to the situation. A depressive experience, on the other hand, is more likely to emerge when assessing patients after the acute phase of the pandemic. The results are in any case consistent with the epidemiological pre-COVID-19 data indicating that anxiety symptoms are more common than depression disturbances in children [30]. The results are also consistent with a recent report the youth with anxiety disorders had a worsening of their symptoms during the COVID-19 pandemic [31].

The parent-completed rating scales (CBCL) were generally consistent with the self-rating scales completed by the children (CDI-2 and MASC-2), revealing a higher than expected rate of internalizing symptoms. These results are consistent with recent reports of high rates of anxiety among children and adolescents during the COVID-19 pandemic. A few studies have also identified an association between the pandemic and depression [32]. About half of the sample had a neuropsychological performance below that expected on attention and/or memory tests. Hospitalization for COVID-19 was not associated with a worse performance on these tests. It should be noted that only a few study participants received oxygen therapy, suggesting that the large majority did not suffer significant hypoxia, which is a risk factor for cognitive impairment [33]. Thus, these results may not necessarily apply to children who suffered more severe respiratory distress from COVID-19. Recent data, however, have shown that even mild COVID-19 can be associated with long-term health sequelae [34].

Given the study design, it cannot be determined whether the observed neuropsychiatric abnormalities were due to a direct action of the virus or the associated inflammatory response, or derived from the psychosocial disruption brought by the pandemic. On one hand, there is evidence that SARS-CoV-2 can enter the CNS through binding to the angiotensin-converting enzyme 2 (ACE2) receptor, which is expressed in both neurons and glia [35], and spread through a variety of possible mechanisms [36]. Through inflammatory response and/or hypoxia, the virus could damage brain structures such as the hippocampus and the basal ganglia, which contain more enzymes involved in inflammatory responses than other areas, thus increasing the risk for deficits in memory, attention, and emotions [37]. On the other hand, the psychological burden of the COVID-19 pandemic on the general population of both adults and children is documented [38], and symptoms such as headache, muscle pain, and concentration difficulties have been reported by COVID-19 negative children [10]. The COVID-19 pandemic was accompanied by drastic physical restrictions and social distancing measures that affected each and every aspect of life [39]. While the number of children and adolescents who were actually infected by the SARS-CoV-2 virus was relatively small, the containment measures with school closure, social distancing, and isolation had a negative impact on mental health and well-being of children and adolescents. Hence, the high rates of anxiety and depression seen in our sample might have been caused by the general social disruption rather than post-infectious sequelae.

The prospective and systematic direct examination of the patients is a strength of this study, which thus expands on previous reports that were mainly based on online surveys. A number of important limitations, however, must be noted. First, the sample size was relatively small. Second, no baseline pre-COVID-19 neuropsychiatric or neurocognitive assessments were available; the study participants were not known for having neuropsychiatric symptoms or academic, social, or family difficulties, and had therefore never been clinically evaluated. As already discussed, a third limitation is represented by the absence of a comparison group of children who lived through the pandemic without however contracting COVID-19, or of a control sample of hospitalized patients with infectious diseases other than COVID-19. Fourth, the time of follow-up was heterogeneous; it was accounted for in the statistical analyses and found not to be associated with the examined outcomes. Finally, the multivariable regression models that were conducted to explore the association between the possible regressors and the neuropsychiatric outcomes of interest had low R^2^, indicating that most of this variance was accounted for by other variables, possibly including pre-COVID-19 inter-subject variability, which was not available for this study.

Further understanding of the long-term implications of COVID-19 can benefit from systematic neurocognitive and neuropsychological assessments of children who did or did not have COVID-19, and for whom pre-COVID assessments are available. This approach, however, would likely limit the inclusion to clinically referred children. Our study focused instead on children who did not have a history of neuropsychiatric problems.

## 5. Conclusions

The high rates of anxiety, depression, headache, and specific neurocognitive deficits including attention, memory, and executive function problems that were found in this sample of children after the resolution of the acute infection support the notion that COVID-19 may have distal neuropsychiatric sequelae. These data can be useful for documenting the type of symptoms possibly associated with long-COVID and highlight the clinical relevance of further evaluating the sequelae of the SARS-CoV-2 infection with controlled investigations.

## Figures and Tables

**Table 1:** Demographics and Clinical Characteristics.

	*N =* 50
Males [n (%)]	28 (56%)
Females [n (%)]	22 (44%)
Hospitalized [n (%)]	35 (70%)
Treated at home [n (%)]	15 (30%)
MIS-C [n (%)]	13 (26%)
Age at the time of the evaluation, years, min–max (median)	8–16 (11.5)
Symptoms during the acute infection [n (%)]:	
Fever	32 (70%)
Cough	13 (26%)
Sore throat	13 (26%)
Abdominal pain	13 (26%)
Skin rash	12 (24%)
Conjunctivitis	11 (22%)
Headache	11 (22%)
Vomiting	11 (22%)
Diarrhea	10 (20%)
Asthenia	10 (20%)
Dyspnea	10 (20%)
Anosmia/ageusia	7 (14%)
Arthromyalgia	7 (14%)
Chest pain	5 (10%)
Cold	20 (10%)
Seizures	1 (2%)
Aphthous stomatitis	1 (2%)
Intensive care	5 (10%)
Oxygen therapy	13 (26%)
Time since the acute infection, months, min–max (median)	1–18 (8)

**Table 2:** Neuropsychiatric Follow-Up Assessments (N = 50).

		In Normal Range		In Borderline/Clinical Range	
		n	%	n	%
SDSC	Total score	36	72	14	28
	Disorders of excessive sleepiness (DES)	35	70	15	30
	Disorders of initiating and maintaining sleep (DIMS)	37	74	13	26
	Sleep breathing disorders (SBD)	44	88	6	12
	Sleep wake transition disorders (SWTD)	44	88	6	12
	Disorders of arousal nightmares (DA)	45	90	5	10
	Sleep hyperhidrosis (SHY)	47	94	3	6
Ped-MIDAS	Disability Grade	44	88	6	12
MASC-2	Total score	26	52	24	48
	Public Performance Fears	22	44	28	56
	Separation Anxiety	24	48	26	52
	Harm Avoidance	25	50	25	50
	Generalized Anxiety Disorder	27	54	23	46
	Social Anxiety	29	58	21	42
	Panic	29	58	21	42
	Obsessions and compulsions	30	60	20	40
	Physical Symptoms	31	62	19	38
	Tense/Restless	33	66	17	34
	Humiliation/Rejection	34	68	16	32
CDI-2	Total score	42	84	8	16
	Ineffectiveness	38	76	12	24
	Functional Problems	40	80	10	20
	Negative Mood	45	90	5	10
	Emotional Problems	46	92	4	8
	Interpersonal Problems	46	92	4	8
	Negative Self-Esteem	46	92	4	8
CBCL	Total score	40	80	10	20
	Internalizing problems	30	60	20	40
	Anxiety	37	74	13	26
	Somatic complaints	38	76	12	24
	Affective Problems	39	78	11	22
	Anxiety Problems	40	80	10	202
	Somatic Problems	40	80	10	0
	Inhibition/Depression	43	86	7	14
	Thought Problems	43	86	7	14
	Externalizing Problems	44	88	6	12
	Attention Problems	47	94	3	6
	Aggressive Behavior	47	94	3	6
	Conduct Problems	47	94	3	6
	Social Problems	47	94	3	6
	Attention Deficit/Hyperactivity	47	94	3	6
	Oppositional Defiant Problems	48	96	2	4
	Rule-breaking Behavior	49	98	1	2
NEPSY-II	Response set	20	40	30	60
	Auditory attention	24	48	26	52
	Inhibition A time	24	48	26	52
	Design fluency	25	50	25	50
	List memory	25	50	25	50
	Visual attention	30	60	20	40
	Inhibition B time	30	60	20	40
	Drawing memory test	30	60	20	40
	Inhibition C time	31	62	19	38
	Animal sorting	32	64	18	36
	Inhibition C combined	33	66	17	34
	Inhibition C error	33	66	17	34
	Inhibition C error	33	66	17	34
	Inhibition A combined	33	66	17	34
	Inhibition B error	36	72	14	28
	Inhibition A error	36	72	14	28
	Inhibition B combined	37	74	13	26
	Word list interference–repetition	40	80	10	20
	Word list interference–re-enactment	40	80	10	20

SDSC: Sleep Disturbance Scale for Children; PedMIDAS: Pediatric Migraine Disability Assessment; MASC-2: Multidimensional Anxiety Scale for Children 2; CDI-2: Children Depression Inventory 2; CBCL: Child Behavior Checklist.

**Table 3:** Evaluation of possible predictors of anxiety (MASC-2), depression (CDI-2), internalizing (CBCL), sleep (SDSC), and neurocognitive disturbances (rankbased estimates of regression models)].

Multidimensional Anxiety Scale for Children—2nd edition (MASC-2)					
	Estimate	Std. Error	t	*p*	
Sex [Female]	−2.0827737	3.528333	−0.5903	0.55808	
Age	−0.3413707	0.710367	−0.4806	0.63327	
Hospitaliz [yes]	3.078022	4.303286	0.7153	0.47831	
MIS-C [yes]	−11.4110832	4.707216	−2.4242	**0.01962**	
PedMidas ^2^	2.08062	4.452401	0.4673	0.64264	
Time ^1^	−0.0029624	0.014049	−0.2109	0.83399	
R^2^ = 0.12					
**Child Depression Inventory 2nd edition (CDI-2)**					
	**Estimate**	**Std. Error**	**t**	***p***	
Sex [Female]	−0.2752695	1.985363	−0.1386	0.89037	
Age	0.181751	0.399717	0.4547	0.65161	
Hospital. [yes]	4.479153	2.421422	1.8498	0.07122	
MIS-C ^1^ [yes]	−2.4021302	2.64871	−0.9069	0.36951	
PedMidas ^2^	2.280707	2.505328	0.9103	0.36772	
Time ^3^	−0.0054655	0.007905	−0.6914	0.49303	
R^2^ = 0.15					
**Child Behavior Checklist (CBCL) Internalizing Symptoms**					
	**Estimate**	**Std. Error**	**t**	***p***	
Sex [Female]	−0.836063	3.220694	−0.2596	0.7964	
Age	−0.392845	0.648429	−0.6058	0.5478	
Hospital. [yes]	3.314423	3.928078	0.8438	0.4035	
MIS-C ^1^ [yes]	−2.379739	4.296789	−0.5538	0.5826	
PedMidas ^2^	6.128051	4.064191	1.5078	0.1389	
Time ^3^	−0.013699	0.012824	−1.0683	0.2914	
R^2^ = 0.09					
**Sleep Disturbance Scale for Children (SDSC)**					
	**Estimate**	**Std. Error**	**t**	***p***	
Sex [Female]	4.037301	2.90745	1.3886	0.1723	
Age	0.55399	0.597811	0.9267	0.3594	
Hospital. [yes]	1.992369	3.488743	0.5711	0.571	
MIS-C ^1^ [yes]	−2.137154	3.816479	−0.5600	0.5785	
PedMidas ^2^	5.333019	3.61232	1.4763	0.1473	
Time ^3^	−0.012149	0.012516	−0.9707	0.3373	
R^2^ = 0.11					
**Neuropsychological Assessment (NEPSY-II)**					
***Visual attention***					
	**Estimate**	**Std. Error**	**t**	***p***	***p* adjusted** ^4^
Sex [Female]	−0.5879919	1.018448	−0.5773	0.56672	0.95
Age	−0.5155826	0.205046	−2.5145	0.01574	0.15
Hospital. [yes]	−0.9192230	1.242137	−0.7400	0.4633	0.95
MIS-C^1^ [yes]	−0.0055350	1.35873	−0.0041	0.99677	0.99
PedMidas ^2^	−0.8583192	1.285178	−0.6679	0.50779	0.95
Time ^3^	0.001186	0.004055	0.2925	0.77133	0.95
R^2^ = 0.18					
**Graphic fluency**					
	**Estimate**	**Std. Error**	**t**	***p***	***p* adjusted** ^4^
Sex [Female]	−0.0806714	1.058304	−0.0762	0.9396	0.99
Age	−0.4914407	0.211424	−2.3244	0.025011	0.15
Hospital. [yes]	2.147341	1.291644	1.6625	0.103862	0.41
MIS-C1 [yes]	−0.3787250	1.412905	−0.2680	0.789975	0.95
PedMidas ^2^	0.644733	1.32159	0.4878	0.628196	0.95
Time ^3^	0.001579	0.004187	0.3771	0.708024	0.95
R^2^ = 0.19					

^1^ MIS-C: Multisystem Inflammatory Syndrome in Children. ^2^ PedMidas: Pediatric Migraine Disability Assessment. ^3^ Days since the acute COVID-19 infection (duration of follow-up). ^4^ Values corrected according to the Benjamini-Hochberg method. Significant *p* values (<0.05) are bolded.

## Data Availability

Requests for the data can be addressed to the authors.
